# Age-Dependent Metabolomic Profile of the Follicular Fluids From Women Undergoing Assisted Reproductive Technology Treatment

**DOI:** 10.3389/fendo.2022.818888

**Published:** 2022-02-16

**Authors:** Yun Huang, Mixue Tu, Yuli Qian, Junyan Ma, Lifen Chen, Yifeng Liu, Yiqing Wu, Kai Chen, Juan Liu, Yanyun Ying, Yao Chen, Yinghui Ye, Lanfeng Xing, Fang Zhang, Yanjun Hu, Runjv Zhang, Ye Chun Ruan, Dan Zhang

**Affiliations:** ^1^ Key Laboratory of Reproductive Genetics (Ministry of Education) and Department of Reproductive Endocrinology, Women’s Hospital, Zhejiang University School of Medicine, Hangzhou, China; ^2^ Key Laboratory of Women’s Reproductive Health Research of Zhejiang Province and Department of Reproductive Endocrinology, Women’s Hospital, Zhejiang University School of Medicine, Hangzhou, China; ^3^ Department of Biomedical Engineering, Faculty of Engineering, The Hong Kong Polytechnic University, Hong Kong, Hong Kong SAR, China

**Keywords:** ovarian aging, follicular fluid, metabolomics, biomarkers, assisted reproductive technology, LC-MS, GC-MS

## Abstract

Female fertility declines with age, and this natural variation culminates in reproductive senescence. Human follicular fluids are rich in low-molecular weight metabolites which are responsible for the maturation of oocytes. The metabolomic approaches are powerful tools to study biochemical markers of oocyte quality in the follicular fluids. It is necessary to identify and quantify the reliable metabolites in follicular fluids reflecting oocyte developmental potential. The goal of this study is to conduct a metabolomic analysis of the follicular fluids in women of different ages and study the metabolomic profile of the follicular fluids in relationship with oocyte quality in assisted reproductive technology (ART) treatment. A total of 30 women seeking for ART treatment at the Women’s Hospital, Zhejiang University School of Medicine from October 2014 to April 2015 were recruited for the present study. Fifteen women aged from 39 to 47 were grouped as advanced maternal age, and the other 15 women aged from 27 to 34, as young controls. Ovarian stimulation and oocyte retrieval were conducted using a regular protocol involving mid-luteal pituitary down-regulation and controlled ovarian stimulation. Follicular fluids from mature follicles were collected and centrifuged for analyses. Liquid Chromatography-Mass Spectrometry (LC-MS) and Gas Chromatography-Mass Spectroscopy (GC-MS) were used to perform the quantitative metabolomic analysis. The follicular fluid levels of 311 metabolites and the metabolic significance were assessed. 70 metabolites showed significant differences between women with young and advanced ages. Follicular fluids from women with advanced age showed significantly higher levels of creatine, histidine, methionine, trans-4-hydroxyproline, choline, mevalonate, N2,N2-dimethylguanosine and gamma-glutamylvaline, as compared to those from the young age group. 8 metabolites were found significantly correlated with maternal age positively. Moreover, 3 metabolites were correlated with the number of oocytes retrieved, and 5 metabolites were correlated with cleaved embryo numbers, both negatively. The follicular fluids from women undergoing ART treatment exhibited age-dependent metabolomic profile. Metabolites associated with oocyte quality were identified, suggesting them as potential biomarkers for oocyte maturation and ART outcomes.

## Introduction

A worldwide growing trend for women to delay motherhood has contributed to the increased incidence of female subfertility as well as the need of fertility treatments for women at advanced ages. It is generally accepted that age alone has an adverse effect on fertility, especially the oocyte quality (1). Maternal age remains the most valuable factor to predict the duration of reproductive life span (2). Age-dependent decline in ovarian function, characterized by decreased oocyte numbers and qualities (3), is attributed to both genetic and environmental factors (4). Follicular fluid, the microenvironment for oocyte development, has been suggested to influence oocyte quality, sperm-mediated oocyte activation as well as early embryo development (5). The human follicular microenvironment contains a complex mixture of steroids, lipids, small peptides, antioxidant enzymes and other metabolites (6), many of which are known to be crucial for oocyte development. For instances, the follicular level of anti-Mullerian hormone was reported to be correlated with oocyte developmental competence during *in vitro* fertilization (7). Lipogenesis and lipolysis play an important role in providing sufficient energy source during oocyte maturation (8, 9). Redox homeostasis is essential to oocyte maturation (10, 11) and its disturbance is involved in the pathogenesis of reproductive disorders (12), such as endometriosis and polycystic ovarian syndrome (13, 14).

Metabolite changes can reflect important maternal biological physiology. A recent study showed that identification of blood metabolites in pregnant women could accurately predict gestational age (15). Metabolomic analysis of follicular fluids is therefore believed be to a good approach to assess oocyte and embryo qualities (16). Levels of metabolites in the follicular fluids were reported to be useful to predict the gamete development potential and select embryos capable of developing into the early cleavage stage (17, 18). However, it remains unknown whether and how the metabolic profile of the follicular fluids would change as the age advances, affecting oocyte maturation or their later development. In the present study, we collected follicular fluid from 30 women of different ages (from 27 to 47 years old) undergoing assisted reproductive technology (ART) treatment for metabolomic analysis. Women of advanced and young ages were compared for their follicular metabolic profiles. Correlation analysis between follicular fluid levels of various metabolites and quantity and quality of oocytes was also performed.

## Patients and Methods

### Woman Subjects and Ethical Approval

Women seeking for ART treatment at the Women’s Hospital, Zhejiang University School of Medicine from October 2014 to April 2015 were recruited for the present study. Fifteen women aged from 39 to 47 were grouped as advanced maternal age, and the other 15 women aged from 27 to 34, as young controls. All the procedures were approved by the Institutional Ethics Committee of Women’s Hospital, Zhejiang University School of Medicine. All participants gave their written informed consents. The inclusion criterion for women seeking for ART treatment was women with tubal factor infertility. Exclusion criteria of subjects were as follows: women who applied for pre-implantation genetic testing (PGT), women with chronic hypertension, heart disease, diabetes, or donor oocyte/embryo recipient cycles.

### Collection of Follicular Fluids

Ovarian stimulation and oocyte retrieval were conducted using a regular protocol involving mid-luteal pituitary down-regulation and controlled ovarian stimulation as we previously reported (19). Oocytes were retrieved by ultrasound-guided transvaginal follicular aspiration. In each woman, the follicular fluid from a mature follicle (18-20 mm in diameter) was aspirated and collected individually into one tube before the oocyte was retrieved. The follicular fluid was immediately transported to the laboratory at room temperature and centrifuged at 2000 rpm for 10 minutes to remove erythrocytes and leukocytes before the supernatant was collected and stored at -80°C until further analysis. Mass spectrometry analysis was performed within one year after sample collection.

### Specimen Processing and Metabolomics Analyses

Each follicular fluid sample was accessioned into the Laboratory Information Management System (LIMS) and assigned by the LIMS a unique identifier that was associated with the original source identifier only. This identifier was used to track all sample handling, tasks, and results. Samples were prepared using the automated MicroLab STAR^®^ system from Hamilton Company. A recovery standard was added prior to the first step in the extraction process. Proteins were precipitated with methanol under vigorous shaking for 2 minutes (Glen Mills GenoGrinder 2000) followed by centrifugation. The resulting extract was divided into five fractions for: 1) ultra-performance liquid chromatography (UPLC) with positive ion mode electrospray ionization, 2) UPLC with negative ion mode electrospray ionization, 3) Liquid Chromatography (LC) polar platform, 4) Gas Chromatography-Mass Spectroscopy (GC-MS), and 5) backup. Samples were placed briefly on a TurboVap^®^ (Zymark) to remove the organic solvent. For LC, the samples were stored overnight in liquid nitrogen before preparation for analysis. For GC, each sample was dried by vacuum for overnight before analysis.

**Figure 1:**
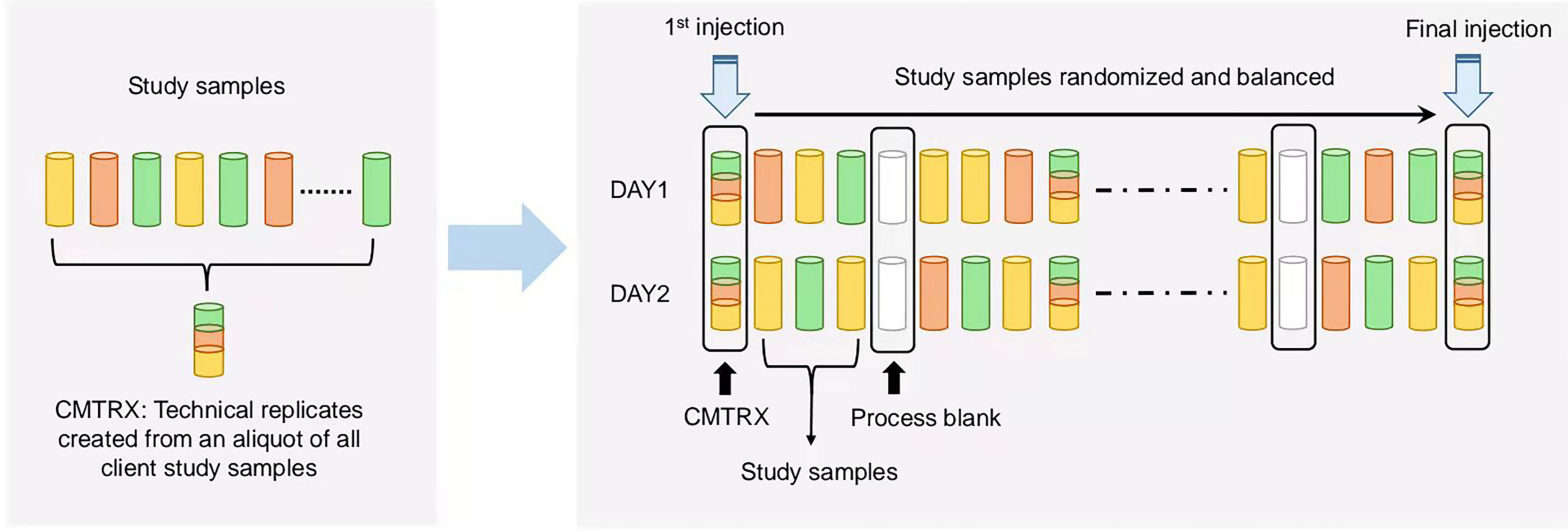
Technical flowchart of sample preparation for mass spectrometry analysis. A small aliquot of each study sample (colored cylinders) was pooled to create a replicate sample (multicolored cylinder), which was then injected periodically throughout the process. For studies spanning multiple days, a data normalization step was performed to correct variation resulting from instrument inter-day tuning differences. Variability among consistently detected biochemical was used to calculate an estimate of overall process variability.

Several types of controls were used in concert with the experimental samples: 1) a pooled matrix sample generated by taking a small volume of each experimental sample served as a technical replicate throughout the data set, 2) extracted water samples served as process blanks, and 3) a cocktail of internal standards that were carefully chosen not to interfere with the measurement of endogenous compounds. Instrument variability was determined by calculating the median relative standard deviation (RSD) for the standards that were added to each sample prior to injection into the mass spectrometers. Overall process variability was determined by calculating the median RSD for all endogenous metabolites present in 100% of the pooled matrix samples. Experimental samples were randomized across the platform run with internal standards samples spaced evenly among the injections, as outlined in **Figure 1**. For studies spanning multiple days, a data normalization step was performed to correct variation resulting from instrument inter-day tuning differences. Essentially, each compound was corrected in run-day blocks by registering the medians to equal one (1.00) and normalizing each data point proportionately.

### Mass Spectrometry Analysis

For LC-MS analysis, the sample extract was dried then reconstituted in acidic or basic LC-compatible solvents, each of which contained 12 or more injection standards at fixed concentrations. One aliquot was analyzed using acidic positive ion-optimized conditions and the other using basic negative ion optimized conditions in two independent injections using separate dedicated columns (Waters UPLC BEH C18-2.1×100 mm, 1.7 μm). Extracts reconstituted in acidic conditions were gradient eluted using water and methanol containing 0.1% formic acid, while the basic extracts, which also used water or methanol, contained 6.5 mM ammonium bicarbonate.

The samples destined for analysis by GC-MS were dried under vacuum for a minimum of 18 hours prior to being derivatized under dried nitrogen using bistrimethyl-silyltrifluoroacetamide. Derivatized samples were separated on a 5% diphenyl or 95% dimethyl polysiloxane fused silica column with helium as carrier gas and a temperature ramp from 60°C to 340°C in a 17.5 minutes period. Samples were analyzed on a Thermo-Finnigan Trace DSQ fast-scanning single-quadrupole mass spectrometer using electron impact ionization (EI) and operated at unit mass resolving power.

### Statistical Analysis

The continuous variables of demographics and clinical outcomes were summarized as mean ± standard deviation (SD). Differences of continuous variables across groups were tested with the unpaired Student’s t-test. The principal component analysis (PCA) was applied for ground discrimination. The levels of 311 metabolites between the advanced age group and young control group were separately compared by using the t-test. To account for the multiple comparisons, the Hochberg and Benjamini false discovery rate (FDR) adjusted *p*-value (i.e., *q*-value) less than 0.05 was applied to select the statistically differentially expressed metabolites between the two groups. The fold change in metabolites was calculated as the ratio of metabolite levels in the advanced age group to the young control group. The Pearson correlation was used to analyze the correlations between each identified metabolite (*q*-value < 0.05) and maternal age, number of oocytes retrieved and number of cleaved embryos. The heatmap was used to intuitively distinguish the metabolites between the two groups. In addition, the association of each identified metabolite with maternal age, number of oocytes retrieved and number of cleaved embryos was estimated with the linear regression model adjusting for the basal FSH levels which were found to be significantly different between the advanced age group and young group. Data analyses were conducted with R version 4.0.3.

## Results

### Participant Characteristics and ART Outcomes

Basic characteristics of the 15 women of advanced (39-47 years old) maternal age and of the other 15 young (27-34 years old) maternal age as controls were listed in **Table 1**. The two groups showed no statistically significant differences in body mass index (BMI), duration of infertility, number of previous cycles received, basal serum levels of luteinizing hormone (LH), estradiol (E_2_), prolactin (Prl), progesterone (P), testosterone (T), total gonadotropin dose, duration of stimulation and endometrial thickness on the day of human chorionic gonadotropin (hCG) administration. However, the group of advanced age showed significantly reduced antral follicle count (*p* = 0.001) with higher basal follicle stimulating hormone (FSH) level (*p* = 0.008), as compared to the young control group. The number of oocytes retrieved (*p* < 0.001), 2PN (two pronuclei) embryos (*p* = 0.001) and cleaved embryos (*p* = 0.001), indicating ART treatment outcomes, were significantly lower in the advanced age group compared to the young control group.

**Table 1:** Baseline characteristics and ART outcomes of the participants included in the study.

Clinical parameters, mean ± SD	Control	Advanced age	*p-value* ^a^
	(n = 15)	(n = 15)	
Maternal age (years)	29.93 ± 2.31	42.27 ± 2.43	**<0.001**
BMI (kg/m²)	22.14 ± 4.04	23.05 ± 2.36	0.456
Duration of infertility (years)	3.20 ± 2.57	3.73 ± 2.79	0.590
Number of previous cycles received (n)	1.33 ± 0.49	1.87 ± 1.19	0.119
Antral follicle count (n)	14.71 ± 5.27	9.07 ± 2.94	**0.001**
Basal FSH (IU/L)	5.48 ± 1.41	8.01 ± 2.85	**0.008**
Basal LH (IU/L)	4.24 ± 3.14	3.81 ± 1.18	0.637
Basal E2(pmol/L)	125.67 ± 65.78	144.39 ± 85.54	0.532
Basal Prl (mIU/L)	19.89 ± 11.45	14.67 ± 6.71	0.200
Basal P (nmol/L)	1.92 ± 0.89	2.38 ± 1.29	0.300
Basal T (nmol/L)	0.84 ± 0.46	0.68 ± 0.43	0.409
Total gonadotropin dose (IU)	2130.83 ± 778.29	2775.00 ± 1027.51	0.067
Duration of stimulation (days)	10.33 ± 1.84	9.86 ± 2.60	0.571
Endometrial thickness on day of hCG (cm)	1.02 ± 0.21	1.07 ± 0.26	0.595
Number of oocytes retrieved (n)	14.60 ± 6.56	6.33 ± 4.72	**<0.001**
Number of 2PN embryos (n)	7.33 ± 3.85	3.13 ± 2.53	**0.001**
Number of cleaved embryos (n)	6.80 ± 3.99	2.60 ± 2.16	**0.001**

ART, assisted reproductive technology; SD, standard deviation; BMI, body mass index; FSH, follicle stimulating hormone; LH, luteinizing hormone; E2, estradiol; Prl, prolactin; P, progesterone; T, testosterone; hCG, human chorionic gonadotropin; 2PN, two pronuclei.

^a^Characteristics between the control group and advanced age group were compared with t-test. The p-values in bold text mean statistically significant (p < 0.05).

### Metabolomic Profiling of Follicular Fluids in Women With Advanced and Young Ages

**Figure 2:**
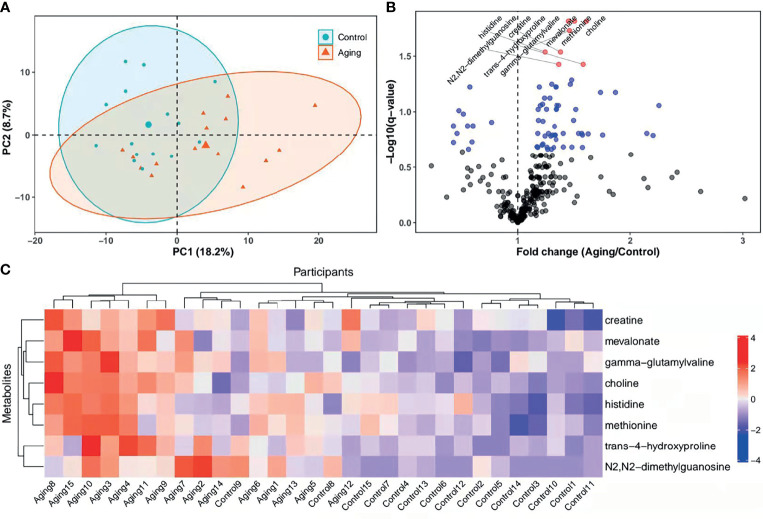
PCA score plots, heatmap and levels of metabolites in different groups. **(A)**, PCA plots and 2D clustering plots describing the trend of separation between the two groups. The red and green points mean the women with advanced and young ages, respectively. **(B)**, Levels of each metabolite between the advanced age group and young control group were tested with unpaired Student’s t-test. The *p*-values of t-test were then adjusted with the FDR method to obtain *q*-values in consideration of multiple comparisons. Fold change was calculated as the ratio of mean of metabolites in the advanced age group to the young control group. In the volcano plot, -log10 (*q*-value) (*y-axis*) was plotted against the fold change (*x-axis*). The metabolites with *p* ≥ 0.05, *p* < 0.05 and *q* < 0.05 were shown with black, blue and red points, respectively. **(C)**, Heatmap of the hierarchical clustering analysis. The selected metabolites (*y-axis*) for each participant (*x-axis*) were presented. Standardized levels of metabolites were shown in different colors. PCA, principal component analysis; 2D, two-dimensional; PC1, first principal component; PC2, second principal component.

The follicular fluids collected from women with advanced or young age were analyzed through mass spectrometry as illustrated in **Figure 1**. Results of PCA showed a separation tendency between the two groups (**Figure 2A**). The volcano plot was applied to visualize the metabolomic profiling of follicular fluids in women with advanced and young ages (**Figure 2B**). A total of 311 metabolites in the follicular fluids were detected, of which 70 had *p* < 0.05 and 8 had *q* < 0.05 when comparing the metabolite levels between the two groups (**Figure 2B** and **Supplementary Table S1**). Among them, biomarkers associated with lipid metabolism, amino acid metabolism and redox homeostasis were identified. In particular, we found that levels of the eight identified metabolites [four amino acids (i.e., creatine, histidine, methionine and trans-4-hydroxyproline), two lipids (mevalonate and choline), one nucleotide (N2,N2-dimethylguanosine) and one peptide (gamma-glutamylvaline)] were significantly higher in the advanced age group compared with the young age group, with fold changes ranging from 1.24 to 1.62. As shown in the heatmap (**Figure 2C**), compared with young women, those with advanced age had higher levels of the selected metabolites.

### Correlations Between Metabolites and Maternal Age, Number of Oocytes Retrieved and Number of Cleaved Embryos

**Figure 3:**
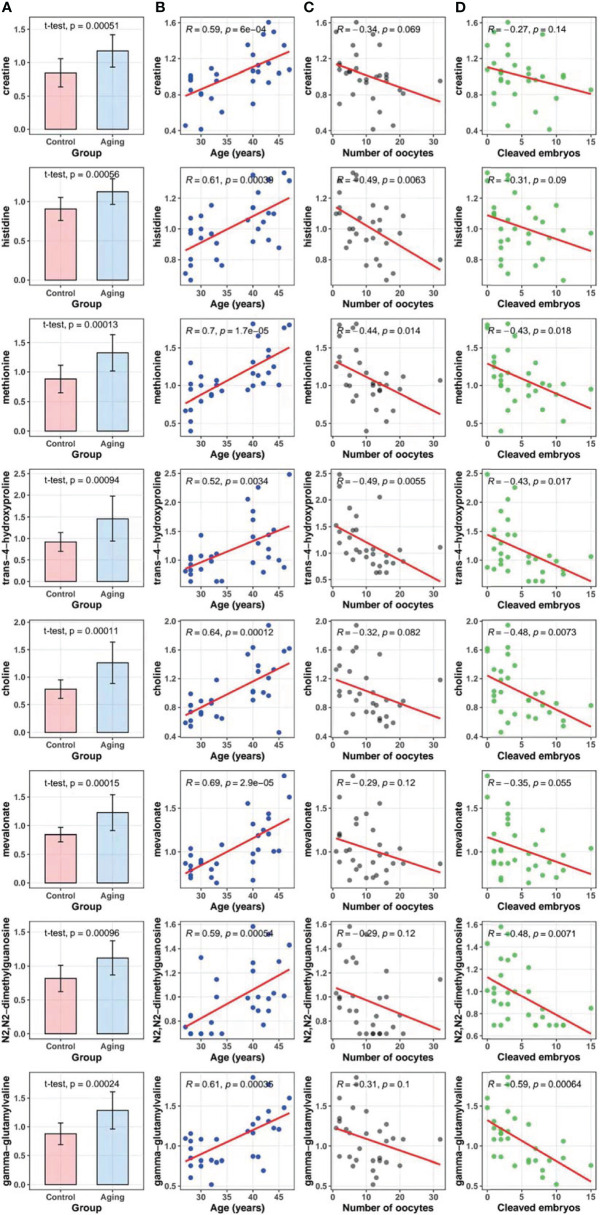
Metabolites in follicular fluids between the advanced age group and the young age group, and correlation tests of maternal age, the number of oocytes retrieved, and cleaved embryo numbers with metabolites. **(A)**, Comparisons of the selected metabolites in follicular fluids between the advanced maternal age group and the young control group (t-test). **(B)**, Correlations between metabolites and maternal age (Pearson correlation test). **(C)**, Correlations between metabolites and the number of oocytes retrieved (Pearson correlation test). **(D)**, Correlations between metabolites and cleaved embryo numbers (Pearson correlation test).

To exam possible effect of these age-dependently changed metabolites on ovarian function and ART outcomes, we did correlation tests for each of them with the number of retrieved oocytes and cleaved embryos, respectively. Our data indicated that levels of all the eight selected metabolites from follicular fluids were higher in women with advanced age compared to the young ones (*p* < 0.05) (panel A in **Figure 3**), and were significantly positively correlated with maternal age among all the participants [coefficients of Pearson correlation (*R*) > 0, *p* < 0.05] (panel B in **Figure 3**). Besides, the levels of amino acid metabolites including histidine (*R* = -0.49, *p* = 0.006), methionine (*R* = -0.44, *p* = 0.014) and trans-4-hydroxyproline (*R* = -0.49, *p* = 0.006) were significantly correlated with decreased number of oocytes retrieved (panel C in **Figure 3**). Correlations between number of oocytes retrieved and the remaining metabolites of creatine, choline, mevalonate, N2,N2-dimethylguanosine and gamma-glutamylvaline were negative but without statistical significance (*R* < 0, *p* > 0.05). The number of cleaved embryos was significantly negatively correlated with five identified metabolites including methionine (*R* = -0.43, *p* = 0.018), trans-4-hydroxyproline (*R* = -0.43, *p* = 0.017), choline (*R* = -0.48, *p* = 0.007), N2,N2-dimethylguanosine (*R* = -0.48, *p* = 0.007) and gamma-glutamylvaline (*R* = -0.59, *p* < 0.001), but not for creatine, histidine and mevalonate (*R* < 0, *p* > 0.05) (panel D in **Figure 3**).

### Associations of Metabolites With Maternal Age, Number of Oocytes Retrieved and Number of Cleaved Embryos

**Figure 4:**
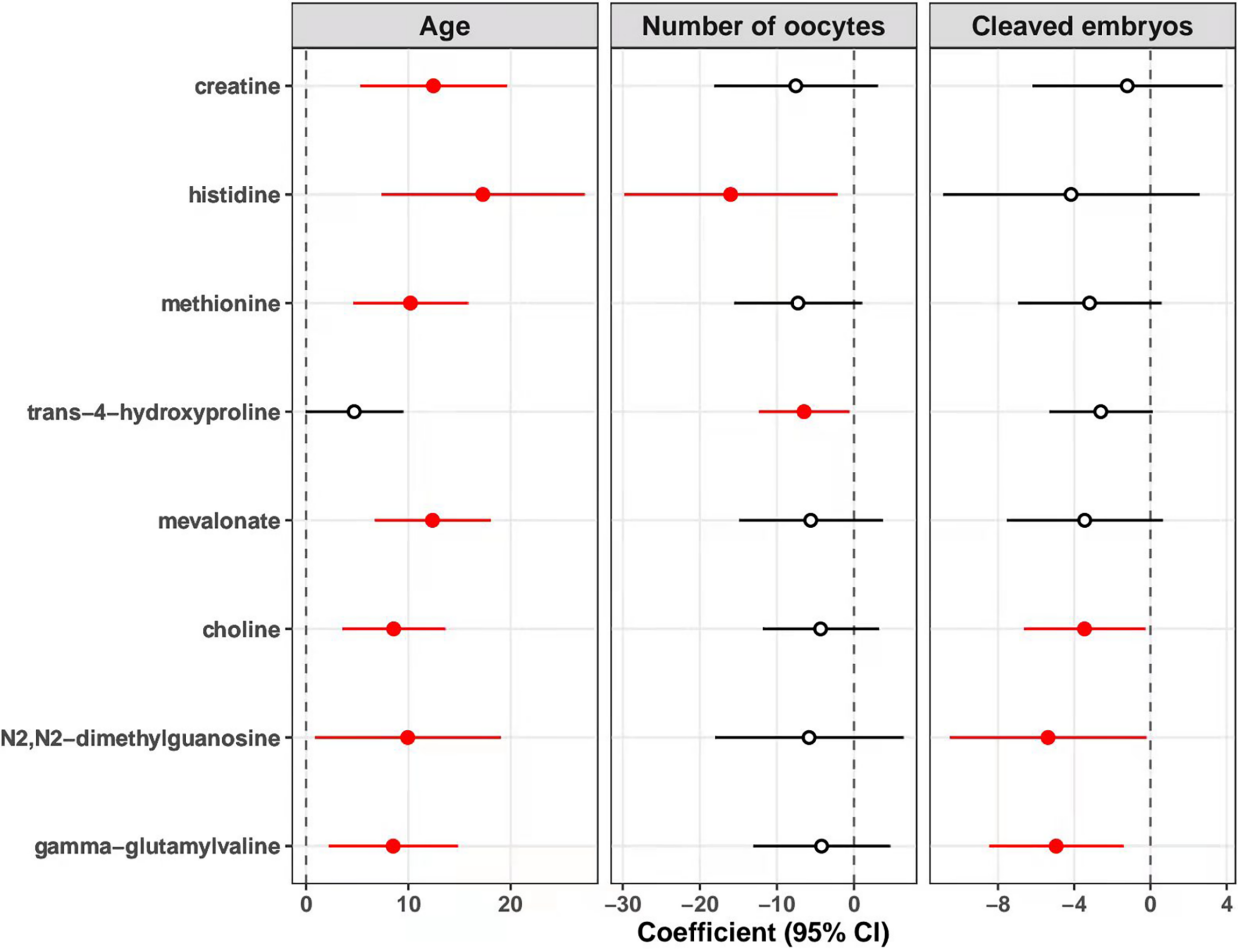
Associations of metabolites with age, number of oocytes retrieved, and cleaved embryo numbers. The association of each identified metabolite with maternal age, number of oocytes retrieved, and number of cleaved embryos was estimated with linear regression model adjusting for the levels of basal FSH. The points and horizontal lines represent the coefficients and 95% confidence intervals (CI) of metabolites, respectively. The solid points in red mean statistically significant (*p* < 0.05). FSH, follicle stimulating hormone.

As shown in **Figure 4**, levels of creatine in follicular fluids were significantly associated with increased age [coefficients of linear regression model (β) = 12.45, *p* = 0.001]. Similarly, we found that the metabolite-age association was significantly positive for the other six metabolites including histidine, methionine, mevalonate, choline, N2,N2-dimethylguanosine and gamma-glutamylvaline (all *p* < 0.05), but not for trans-4-hydroxyproline (*p* = 0.053). Besides, levels of histidine (β = -16.01, *p* = 0.025) and trans-4-hydroxyproline (β = -6.48, *p* = 0.031) were associated with decreased number of oocytes retrieved. Levels of choline (β = -3.46, *p* = 0.033), N2,N2-dimethylguanosine (β = -5.38, *p* = 0.041) and gamma-glutamylvaline (β = -8.46, *p* = 0.008) showed significantly inverse association with number of cleaved embryos.

## Discussion

The present metabolomic profiling of the follicular fluids from women undergoing ART treatment has revealed age-dependent metabolism in human follicles, including age-dependent changes in biochemical molecules or biomarkers related to redox homeostasis, amino acid and lipid metabolism. Interestingly, significant correlations between levels of the identified metabolites and oocyte number and cleaved embryos were also observed, suggesting these metabolites as potential biomarkers for oocyte maturation and ART outcomes. This study can promote the current understanding of the age-dependent metabolomic profile of human follicular fluids, may provide new insights into the endocrinology of aging, and offer future directions for ovarian function and reproductive aging research. Our results will be helpful for noninvasive prediction of embryonic developmental potential and systematic evaluation system of ART outcomes.

Ovary is the most important reproductive endocrine organ in women. The reproductive and endocrine functions of ovary decline with aging, mainly depending on the number and quality of ovarian follicles (20). Some metabolites decreased in follicular fluids, like advanced glycation end-products, have been found to improve oocyte developmental potential (21). The steroid metabolism in the follicles seems to be tightly associated with ovarian function and oocyte quality. As expected, decreases in the production of several steroids (i.e., 21-hydroxypregnenolone disulfate, pregnanediol-3-glucuronide and cortisol) were observed in the advanced maternal age group, suggesting age-related decline in these steroids production. However, increases in follicular progesterone, 17-alpha-hydroxyprogesterone and dehydroepiandrosterone (DHEA) were seen in the advanced age group, which is possibly due to increased cholesterol metabolism as suggested by significantly elevated follicular mevalonate.

**Figure 5:**
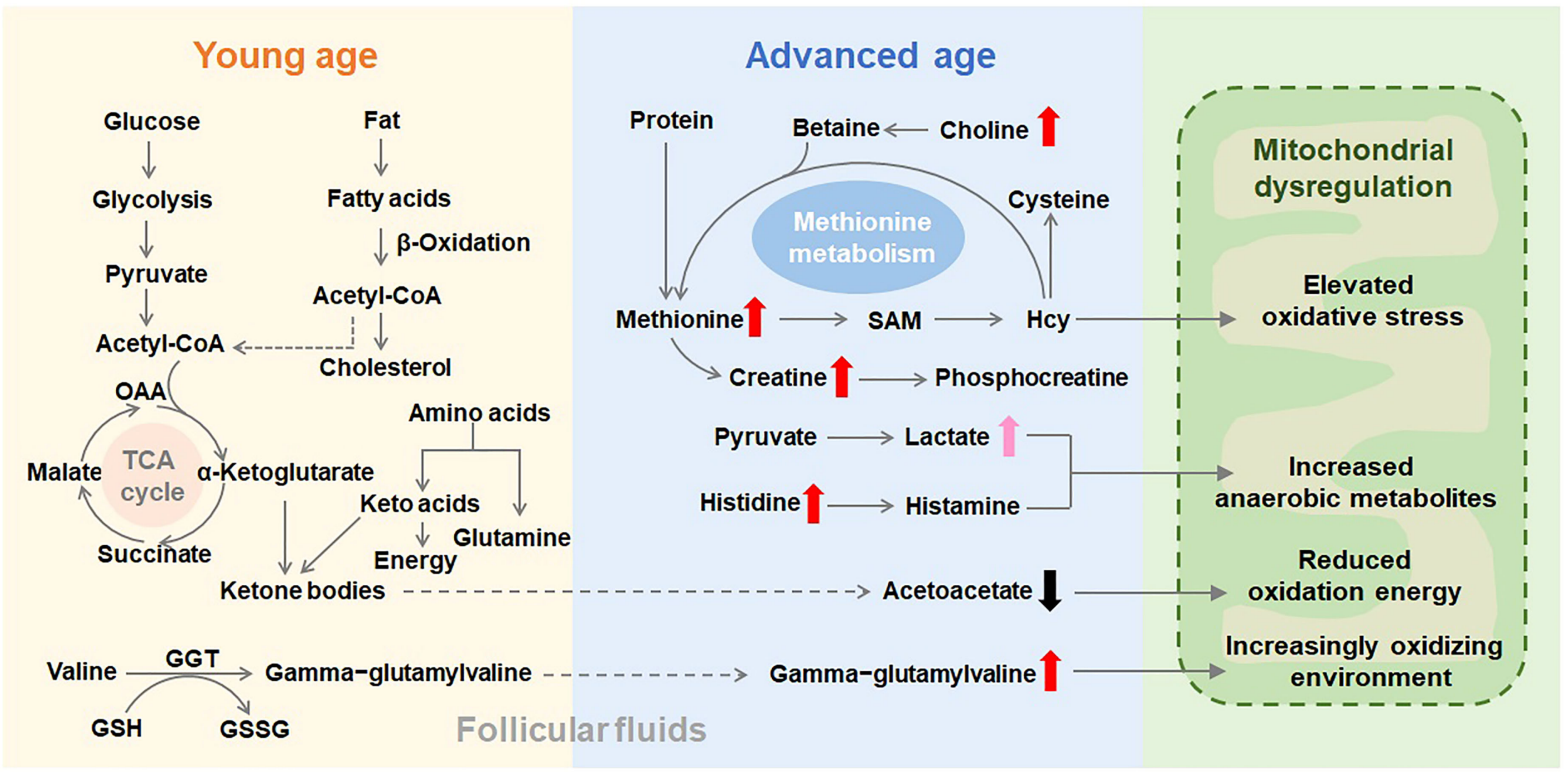
Schematic representation of the metabolic pathways that are putatively altered in follicular fluids as age advances. Thick and short arrows indicate the variation in metabolite concentrations in follicular fluids from women with advanced age compared with their concentrations in the young age group. A dysfunctional mitochondrial metabolism could explain the observed accumulation of identified metabolites in the follicular fluids of advanced age women. SAM, S-adenosylmethionine; Hcy, homocysteine; GSH, glutathione; GSSG, oxidized glutathione; GGT, gamma-glutamyltransferase; OAA, oxaloacetic acid; TCA cycle, tricarboxylic acid cycle.

Glucose and fatty acids are vital energy substrates for many cell activities, and fatty acid β-oxidation is crucial for the quality of oocyte and embryo development competence (22, 23). An increase in lactate indicates a metabolic shift from mitochondrial oxidative phosphorylation to glycolysis. Although follicular glucose level didn’t show difference between the two groups of different ages, lactate level in the follicular fluid was increased in advanced age group suggesting increased anaerobic glycolysis. Lipid metabolism disorder was proved to be associated with female reproductive and endocrine functions (24). The current literature indicates that higher lipolysis and lower lipogenesis enhance the delivery of lipid metabolites (25). Levels of fatty acids were changed as a class (**Supplementary Table S1**), especially increases in several acylcarnitines (i.e., octanoylcarnitine and decanoylcarnitine) may suggest decreasing β-oxidative use, which is supported by declines in the ketone body acetoacetate. Finally, increases in deoxycarnitine could be consistent with declining demand for use in β-oxidation. A decrease in ketogenic use has previously been associated with aging in rodents (26), which is consistent with the presently observed energetics shift in humans. Choline can modulate the expression of a class of proteins involved in the metabolism of ketone bodies and fatty acids (27). It is transported into mitochondria, where choline metabolism leads to increases in oxygen consumption and ATP production (28). Mitochondrial metabolic imbalance may lead to decreased choline utilization, resulting in its accumulation in follicles. Follicular fluids of women with advanced age display lower expression of proteins involved in mitochondrial function. In turn, this leads to lower availability of mitochondria-derived energy sources for oocyte maturation. Mitochondrial metabolism is tightly associated with male spermatogenesis (29), female fertility and the fate of ovarian follicles (30). Our results indicate a mitochondrial dysregulation in the advanced age group (**Figure 5**), with a modified balance between β-oxidation and glycolysis that could affect the fertility of women with advanced age.

Glutathione serves several vital functions such as acting as radical scavenger, antioxidant and modulating critical cell processes including immune function (31). There was experimental evidence that glutathione levels declined with age (32). The present study observed that glutathione metabolic status was altered in the advanced age group. Though oxidized glutathione was not significantly changed, increases in gamma-glutamyl amino acids (i.e., gamma-glutamylleucine, gamma-glutamyltyrosine and gamma-glutamylvaline) could suggest an increasingly oxidizing environment. Oxidative stress could have a deleterious effect on mitochondrial function and oocyte quality (33), while 5-oxoproline may reduce non-enzymatic antioxidant defenses (34). An increase in 5-oxoproline (also known as L-pyroglutamic acid) is consistent with increased exchange of gamma-glutamyl amino acids to regenerate glutathione. Oxidative stress has been implicated in a variety of aspects of aging (11), including the role of oxidative stress in ovarian aging (35). Age-related oxidative damage to lipid, amino acid and other cellular components may play a role in diminished ovarian function that occur as maternal age advances. It was reported that appropriate treatment with the antioxidant N-acetyl-L-cysteine (NAC) postpones the process of ovarian aging in mice (36).

Significantly increased level of tRNA-specific modified nucleoside (N2,N2-dimethylguanosine) was identified in the advanced age group. N2,N2-dimethylguanosine promotes the folding of intracellular tRNAs toward the classical cloverleaf structure (37). The reversible and dynamic nature of nucleoside modifications identifies these metabolites as candidates to monitor the response of related proteins (38). The altered level of specific nucleoside modifications in women with advanced maternal age, as exemplified by the increased expression of N2,N2-dimethylguanosine, may reflect preferentially expressed tRNAs that harbor them toward the translation of stress response proteins. We observed that the level of methionine was significantly higher in the advanced age group compared with the young age group. After adjusting for the basal FSH level, the metabolite-age association was still significantly positive (**Figure 4**). Methionine is an aliphatic, sulfur-containing amino acid and a precursor of homocysteine (Hcy) and creatine (39). An elevated level of the Hcy has been confirmed to be connected with pregnancy problems (40). The circulating levels of Hcy can be increased by deficiencies of vitamins B_12_ and folic acid, defects in enzymes of the methionine metabolism or by feeding methionine enriched diets (41). Recent research demonstrated that methionine could regulate metabolic processes including lipid metabolism and oxidative stress (42). In our study, bone resorption biomarker trans-4-hydroxyproline was also found age-dependently increased in human follicular fluids. Elevated whole body hydroxyproline production has been confirmed be associated with bone mineral loss (43, 44). Increased expression of this metabolite in advanced age group may help to explain why osteoporosis occurs with aging.

Among the differentially expressed metabolites in follicular fluids, some of them were confirmed to be associated with oocyte number or their developing capacity after fertilization. Age-dependently increased amino acids (i.e., histidine, methionine and trans-4-hydroxyproline) showed strong negative correlation with the number of oocytes retrieved, suggesting these amino acids as crucial factors for oocyte quantity, and their age-dependent increase may account for the declined ovarian function over age. Very interestingly, a group of metabolites associated with amino acid metabolism, not only showed significant correlation with the oocyte number, but also are in significant correlation with the number of cleaved embryos. Cleaved embryo numbers are effective parameters to indicate developing capacity of selected oocytes in ART treatment. Age-dependently increased methionine and trans-4-hydroxyproline were found in negative correlation with the number of cleaved embryos, suggesting that these factors may play critical roles in oocyte development and could serve as potential predicting factors for ART outcomes. There were some limitations in the present study. Although the *post-hoc* statistical powers of all the 8 selected metabolites were higher than 0.80 (data not shown), the limited sample size might lead to low statistical power and attenuated capabilities to identify other influential metabolites.

In conclusion, the follicular fluids from women undergoing ART treatment exhibited age-dependent metabolomic profile. The identification of significantly overexpressed metabolites, especially methionine and trans-4-hydroxyproline, may contribute to find an effective way to optimize oocyte quality and evaluate ART outcomes for women with aging-related infertility. Results of the present study suggest that it is very important to pay close attention to the human follicular microenvironment of women with advanced age. Nevertheless, the ovarian aging process is complicated and it is caused by numerous factors that control cellular life span. Follow-up studies in a similar population with larger cohorts will be necessary to elucidate the alteration of these follicular metabolites. Molecular mechanisms underlying their age-dependent changes as well as their roles in oocyte development await further experimental investigation.

## Data Availability Statement

The original contributions presented in the study are included in the article/**Supplementary Material**. Further inquiries can be directed to the corresponding author.

## Ethics Statement

The studies involving human participants were reviewed and approved by Institutional Ethics Committee of Women’s Hospital, Zhejiang University School of Medicine. The patients/participants provided their written informed consent to participate in this study.

## Author Contributions

DZ and YH designed the study. LC, YW, and KC collected samples. YH, YQ, and MT performed the experiments. JM, YL, and JL participated in data analysis and interpretation. YH, MT, and YQ drafted the initial manuscript. YR, YJH, and LX edited the manuscript. YHY, YYY, and YC contributed to critical discussion. DZ, RZ, and FZ supervised and supported the whole project. DZ provided final approval of the manuscript. All authors read and agreed on the final version of the manuscript.

## Funding

This work was supported by the National Key Research and Development Program of China (No. 2018YFC1005003, 2021YFC2700601, 2021YFC2700402), the National Natural Science Foundation of China (No. 81974224), the Key Research and Development Program of Zhejiang Province (No. 2021C03098), the Natural Science Foundation of Zhejiang Province (No. LZ18H040001, LQ21H040004), and Zhejiang University Education Foundation Global Partnership Fund. The work was supported in part by Y.C. Ruan, and Early Career Scheme by Research Grants Council of Hong Kong (Y.C.R. No.24104517).

## Conflict of Interest

The authors declare that the research was conducted in the absence of any commercial or financial relationships that could be construed as a potential conflict of interest.

## Publisher’s Note

All claims expressed in this article are solely those of the authors and do not necessarily represent those of their affiliated organizations, or those of the publisher, the editors and the reviewers. Any product that may be evaluated in this article, or claim that may be made by its manufacturer, is not guaranteed or endorsed by the publisher.

## Abbreviations

ART, assisted reproductive technology; DHEA, dehydroepiandrosterone; E_2_, estradiol; FDR, false discovery rate; FSH, follicle stimulating hormone; GC-MS, gas chromatography-mass spectroscopy; GC, gas chromatography; Hcy, homocysteine; LC-MS, liquid chromatography-mass spectrometry; LC, liquid chromatography; LH, luteinizing hormone; LIMS, laboratory information management system; P, progesterone; PCA, principal component analysis; PGT, pre-implantation genetic testing; Prl, prolactin; R, coefficients of Pearson correlation; RSD, relative standard deviation; SD, standard deviation; T, testosterone; UPLC, ultra-performance liquid chromatography; hCG, human chorionic gonadotropin; β, coefficients of linear regression model; 2PN, two pronuclei.
