# Supplementary material for: Age-Dependent Metabolomic Profile of the Follicular Fluids From Women Undergoing Assisted Reproductive Technology Treatment
Source: Front Endocrinol (Lausanne). 2022 Feb 16;13:818888. doi: 10.3389/fendo.2022.818888 (PMC8888916; doi:10.3389/fendo.2022.818888)
Supplement: Supplementary file 1 [file DataSheet_1.pdf]

Supplementary Table S1. Differences in metabolite levels in the advanced age group, compared with the young age group.

No.	Pathway	Sub Pathway	PubChem CID	Biochemical name	Fold change (A/C) [a]	Change direction	*p*-value (t-test) [b]	*q*-value (FDR-adjusted) [c]
1	Amino Acid	Alanine and Aspartate Metabolism	5950	alanine	1.48	Up	0.0420	0.2075
2	Amino Acid	Creatine Metabolism	586	creatine	1.38	Up	0.0005	**0.0290**
3	Amino Acid	Glutamate Metabolism	5961	glutamine	1.19	Up	0.0029	0.0598
4	Amino Acid	Glutathione Metabolism	7405	5-oxoproline	1.20	Up	0.0068	0.0877
5	Amino Acid	Glycine, Serine and Threonine Metabolism	673	dimethylglycine	1.26	Up	0.0427	0.2077
6	Amino Acid	Glycine, Serine and Threonine Metabolism	750	glycine	1.32	Up	0.0447	0.2086
7	Amino Acid	Histidine Metabolism	6274	histidine	1.24	Up	0.0006	**0.0290**
8	Amino Acid	Leucine, Isoleucine and Valine Metabolism	6426901	2-methylbutyrylcarnitine (C5)	1.47	Up	0.0019	0.0565
9	Amino Acid	Leucine, Isoleucine and Valine Metabolism	6426851	isovalerylcarnitine	1.54	Up	0.0212	0.1560
10	Amino Acid	Leucine, Isoleucine and Valine Metabolism	6287	valine	1.22	Up	0.0055	0.0854
11	Amino Acid	Lysine Metabolism	5962	lysine	1.34	Up	0.0263	0.1614
12	Amino Acid	Lysine Metabolism	92832	N6-acetyllysine	1.18	Up	0.0439	0.2086
13	Amino Acid	Methionine, Cysteine, SAM and Taurine Metabolism	6137	methionine	1.51	Up	0.0001	**0.0153**
14	Amino Acid	Phenylalanine and Tyrosine Metabolism	6140	phenylalanine	1.18	Up	0.0227	0.1560
15	Amino Acid	Phenylalanine and Tyrosine Metabolism	6057	tyrosine	1.30	Up	0.0020	0.0565
16	Amino Acid	Urea cycle; Arginine and Proline Metabolism	9750	citrulline	1.34	Up	0.0025	0.0598
17	Amino Acid	Urea cycle; Arginine and Proline Metabolism	6262	ornithine	1.27	Up	0.0231	0.1560
18	Amino Acid	Urea cycle; Arginine and Proline Metabolism	145742	proline	1.22	Up	0.0369	0.1946
19	Amino Acid	Urea cycle; Arginine and Proline Metabolism	5810	trans-4-hydroxyproline	1.58	Up	0.0009	**0.0375**
20	Amino Acid	Urea cycle; Arginine and Proline Metabolism	1176	urea	1.52	Up	0.0089	0.0950
21	Carbohydrate	Aminosugar Metabolism	2781043	erythronate*	1.25	Up	0.0081	0.0947

22	Carbohydrate	Fructose, Mannose and Galactose Metabolism	6251	mannitol	1.26	Up	0.0487	0.2168
23	Carbohydrate	Glycolysis, Gluconeogenesis, and Pyruvate Metabolism	612	lactate	1.35	Up	0.0082	0.0947
24	Carbohydrate	Pentose Metabolism	94154	arabitol	1.22	Up	0.0384	0.1990
25	Carbohydrate	Pentose Metabolism	6602431	xylonate	1.48	Up	0.0015	0.0518
26	Cofactors and Vitamins	Tocopherol Metabolism	14985	alpha-tocopherol	1.57	Up	0.0228	0.1560
27	Energy	TCA Cycle	525	malate	1.37	Up	0.0064	0.0877
28	Energy	TCA Cycle	1110	succinate	1.18	Up	0.0086	0.0950
29	Lipid	Carnitine Metabolism	134	deoxycarnitine	1.28	Up	0.0045	0.0752
30	Lipid	Fatty Acid Metabolism (also BCAA Metabolism)	439829	butyrylcarnitine	1.30	Up	0.0480	0.2168
31	Lipid	Fatty Acid Metabolism (also BCAA Metabolism)	107738	propionylcarnitine	1.29	Up	0.0289	0.1648
32	Lipid	Fatty Acid Metabolism(Acyl Carnitine)		cis-4-decenoyl carnitine	1.50	Up	0.0123	0.1194
33	Lipid	Fatty Acid Metabolism(Acyl Carnitine)	10245190	decanoylcarnitine	1.41	Up	0.0413	0.2070
34	Lipid	Fatty Acid Metabolism(Acyl Carnitine)	123701	octanoylcarnitine	1.58	Up	0.0270	0.1614
35	Lipid	Fatty Acid, Branched	12587	isocaproate	1.31	Up	0.0251	0.1614
36	Lipid	Fatty Acid, Dicarboxylate	123979	3-carboxy-4-methyl-5-propyl-2-furanpropanoate (CMPF)	2.15	Up	0.0205	0.1560
37	Lipid	Glycerolipid Metabolism	754	glycerol 3-phosphate	1.24	Up	0.0292	0.1648
38	Lipid	Ketone Bodies	96	acetoacetate	0.46	Down	0.0225	0.1560
39	Lipid	Lysolipid		1-stearoyl-GPI (18:0)	1.17	Up	0.0403	0.2054
40	Lipid	Medium Chain Fatty Acid	8892	caproate (6:0)	1.33	Up	0.0169	0.1351
41	Lipid	Mevalonate Metabolism	439230	mevalonate	1.45	Up	0.0001	**0.0153**
42	Lipid	Phospholipid Metabolism	305	choline	1.62	Up	0.0001	**0.0153**
43	Lipid	Polyunsaturated Fatty Acid (n3 and n6)	445580	docosahexaenoate (DHA; 22:6n3)	1.33	Up	0.0270	0.1614

44	Lipid	Polyunsaturated Fatty Acid (n3 and n6)	446284	eicosapentaenoate (EPA; 20:5n3)	1.74	Up	0.0035	0.0669
45	Lipid	Steroid	6238	17-alpha-hydroxyprogesterone	1.87	Up	0.0037	0.0669
46	Lipid	Steroid	134595	21-hydroxypregnenolone disulfate	0.60	Down	0.0164	0.1341
47	Lipid	Steroid	10634	4-androsten-3beta,17beta-diol disulfate (1)*	1.76	Up	0.0256	0.1614
48	Lipid	Steroid	242332	5alpha-androstan-3beta,17beta-diol disulfate	2.20	Up	0.0278	0.1629
49	Lipid	Steroid		5alpha-pregnan-3alpha,20beta-diol disulfate 1*	0.48	Down	0.0094	0.0976
50	Lipid	Steroid		5alpha-pregnan-3beta,20alpha-diol disulfate	0.52	Down	0.0104	0.1046
51	Lipid	Steroid	66416	beta-estradiol 3-sulfate	2.26	Up	0.0060	0.0877
52	Lipid	Steroid	5754	cortisol	0.77	Down	0.0140	0.1245
53	Lipid	Steroid	5881	dehydroepiandrosterone	1.41	Up	0.0228	0.1560
54	Lipid	Steroid	5757	estradiol	1.58	Up	0.0449	0.2086
55	Lipid	Steroid		pregn steroid monosulfate*	0.57	Down	0.0027	0.0598
56	Lipid	Steroid	123796	pregnanediol-3-glucuronide	0.55	Down	0.0162	0.1341
57	Lipid	Steroid		pregnen-diol disulfate*	0.56	Down	0.0488	0.2168
58	Lipid	Steroid	5994	progesterone	1.34	Up	0.0319	0.1769
59	Nucleotide	Purine Metabolism, (Hypo)Xanthine/Inosine containing	6021	inosine	1.63	Up	0.0238	0.1575
60	Nucleotide	Purine Metabolism, Guanine containing	92919	N2,N2-dimethylguanosine	1.37	Up	0.0010	**0.0375**
61	Nucleotide	Pyrimidine Metabolism, Orotate containing	648	dihydroorotate	1.34	Up	0.0081	0.0947
62	Nucleotide	Pyrimidine Metabolism, Thymine containing	1135	thymine	0.59	Down	0.0341	0.1863
63	Nucleotide	Pyrimidine Metabolism, Uracil containing	445408	5-methyluridine (ribothymidine)	1.22	Up	0.0133	0.1218

64	Nucleotide	Pyrimidine Metabolism, Uracil containing	6029	uridine	1.35	Up	0.0066	0.0877
65	Peptide	Gamma-glutamyl Amino Acid	151023	gamma-glutamylleucine	1.18	Up	0.0127	0.1194
66	Peptide	Gamma-glutamyl Amino Acid	94340	gamma-glutamyltyrosine	1.35	Up	0.0029	0.0598
67	Peptide	Gamma-glutamyl Amino Acid	7015683	gamma-glutamylvaline	1.46	Up	0.0002	**0.0186**
68	Xenobiotics	Chemical	23831	HEPES	0.43	Down	0.0159	0.1341
69	Xenobiotics	Chemical	4766	phenol red	0.43	Down	0.0351	0.1881
70	Xenobiotics	Food Component/Plant	222285	erythritol	1.24	Up	0.0046	0.0752

[a] Fold change is calculated as the mean levels of metabolites in the advanced age group, relative to those in the young control group. A/C, the advanced age group/ the young control group.

[b] Levels of metabolites between the advanced age group and young control group were compared with the t-test.

[c] The *q*-value was estimated by the false discovery rate (FDR) approach to take into account the multiple comparisons.
